# Trace amine–associated receptor 1 agonists differentially regulate dopamine transporter function

**DOI:** 10.1016/j.molpha.2025.100064

**Published:** 2025-07-28

**Authors:** Julia K. Huey, Xiao Shi, William E. Schutzer, Aaron Janowsky, Atheir I. Abbas

**Affiliations:** 1 Program in Physiology and Pharmacology, Department of Chemical Physiology and Biochemistry, Oregon Health & Science University, Portland, Oregon; 2 Department of Chemical Physiology and Biochemistry, Oregon Health & Science University, Portland, Oregon; 3 Research Service, Veterans Affairs Portland Health Care System, Portland, Oregon; 4 Department of Psychiatry, Oregon Health & Science University, Portland, Oregon; 5 Department of Behavioral Neuroscience, Oregon Health & Science University, Portland, Oregon

**Keywords:** Trace amine–associated receptor 1, Dopamine transporter, Amphetamine

## Abstract

Trace amine–associated receptor 1 (TAAR1) is a G protein-coupled receptor stimulated by trace amines and amphetamine-like psychostimulants. The TAAR1 agonists RO5166017 and RO5256390 have antidepressant- and anxiolytic-like effects in preclinical models, and the TAAR1/partial 5HT_1A_ agonist ulotaront has been evaluated for its clinical utility as an antipsychotic. Early clinical investigations of ulotaront for treating psychosis in schizophrenia yielded positive endpoints. However, results from phase III clinical trials showed that ulotaront had the same efficacy as placebo. One concern arising from these results is that investigational TAAR1 agonists could be diverse in the mechanisms by which they influence dopamine homeostasis. Thus, we evaluated the pharmacology of TAAR1 agonists RO5166017, RO5256390, and ulotaront at the dopamine transporter (DAT) and TAAR1 to test our hypothesis that there would be differences among agonists in their effects on DAT. We found that RO5166017 and RO5256390 directly bind DAT and inhibit dopamine uptake, while ulotaront did not. In cultured cells and rodent synaptosomes, pretreatment with ulotaront and RO5256390 reduced dopamine uptake by approximately half, while RO5166017 pretreatment increased dopamine uptake by a similar magnitude. In cells, the effects of TAAR1 agonist pretreatment on dopamine uptake were TAAR1-dependent. RO5166017, but not RO5256390 or ulotaront, increased amphetamine-induced dopamine efflux in a TAAR1-dependent manner. Surface biotinylation experiments indicated that RO5166017 pretreatment increased cell-surface DAT by approximately 15% via a TAAR1-dependent mechanism. These findings demonstrate clinically relevant differences in the effects of 3 TAAR1 agonists on DAT.

**Significance Statement:**

This study evaluated the direct and heterologous TAAR1-dependent effects of the TAAR1 agonists RO5166017, RO5256390, and ulotaront at DAT. All 3 affected DAT transport and/or trafficking, with each exhibiting a unique profile of direct and heterologous effects, some of which were TAAR1-dependent. These issues should be considered with therapeutic design and clinical use of TAAR1 agonists.

## 1 Introduction

Trace amine–associated receptor 1 (TAAR1), a G protein-coupled receptor bearing sequence and structural homology with other monoamine receptors, is present in several monoaminergic regions of the brain, including the ventral tegmental area (VTA).^1^, ^2^, ^3^ Endogenously, TAAR1 is stimulated by trace amines, compounds that are structurally similar to biogenic amine neurotransmitters and are present in low concentrations throughout the central nervous system.^4^ Trace amines such as *β*-phenylethylamine and *p*-tyramine have sympathomimetic effects, and structurally similar phenethylamine derivatives such as amphetamine (AMPH) and methamphetamine (METH) are also TAAR1 agonists.^5^ Indeed, many synthetic phenethylamines are potent TAAR1 agonists, and TAAR1-mediated signaling plays an important role in the mechanism of action of these drugs.^3^^,^^6^, ^7^, ^8^ Trace amines are not selective TAAR1 agonists; they are promiscuous ligands with various TAAR1- and non–TAAR1-mediated physiological effects.^9^ Similarly, the drugs of abuse METH and AMPH modulate dopamine (DA) levels via TAAR1- and non–TAAR1-mediated effects on the dopamine transporter (DAT).^8^^,^^10^^,^^11^

In the VTA, TAAR1 modulates dopaminergic transmission, although the precise physiological role of TAAR1 remains unknown. VTA DA neurons from TAAR1 knockout (TAAR1^−/−^) animals have a dramatically increased spontaneous firing rate compared to those of their wildtype counterparts, suggesting that TAAR1 modulates dopaminergic tone, supporting the potential utility of TAAR1 agonists in hyperdopaminergic disorders such as schizophrenia.^12^, ^13^, ^14^ Despite the dramatic differences in basal dopaminergic tone, TAAR1^−/−^ animals do not exhibit functional differences in motor control, coordination, or spontaneous locomotion.^12^^,^^15^ However, behavioral experiments demonstrated that TAAR1^−/−^ mice exhibit increased sensitivity to AMPH, and a clinical study identified a correlation between a TAAR1 polymorphism and drug craving in individuals with METH dependence, illustrating a critical role of TAAR1 in physiological and behavioral responses to psychostimulants.^12^^,^^16^^,^^17^

Because METH and AMPH affect DAT via multiple mechanisms, it has been difficult to disentangle the role of TAAR1 in influencing DA levels. In 2009, the first TAAR1-specific antagonist, EPPTB (*N*-(3-ethoxyphenyl)-4-pyrrolidin-1-yl-3-trifluoromethyl-benzamide; RO5212773), was reported, and shortly thereafter, several specific TAAR1 agonists were developed.^14^^,^^18^^,^^19^ Two of these agonists, RO5166017 and RO5256390, have been used extensively in preclinical TAAR1 research that has produced much of the currently available data concerning the role of TAAR1 in modulating dopaminergic transmission.^20^^,^^21^ In preclinical animal models, RO5166017 and RO5256390 decrease the spontaneous firing rate of DA neurons and produce antidepressant-, antipsychotic-, and anxiolytic-like effects.^18^^,^^21^^,^^22^ Although these studies demonstrate that TAAR1 signaling affects DA neurons and therefore DA release, comparatively less work has been done to explore whether, like AMPHs, these drugs affect DA levels via DAT.

The discovery of TAAR1-mediated modulation of dopaminergic neurotransmission led to the investigation of TAAR1 agonists as potential therapeutics for various psychiatric disorders including schizophrenia and substance use disorders. Recently, the investigational compound ulotaront, a TAAR1/partial-5HT_1A_ agonist, was reported as a first-in-class (non–D2-interacting) antipsychotic.^21^^,^^23^ Although ulotaront performed well in early clinical investigation, phase III clinical trials demonstrated no differences in clinical efficacy of ulotaront compared to placebo.^24^ One possible explanation of these results is that investigational TAAR1 agonists may be more diverse than initially thought in the mechanisms by which they influence DA homeostasis, and the recent finding that AMPH induces DAT internalization in a TAAR1-dependent manner, thereby reducing DA uptake, raises the question of whether other TAAR1 agonists might have similar effects on DAT trafficking and DA uptake.^8^ As a result, we compared the pharmacology of 3 TAAR1 agonists, RO5166017, RO5256390, and ulotaront, to test our hypothesis that there would be clinically relevant differences in their effects on DAT. In this report, we assess the ability of these 3 TAAR1 agonists to bind to DAT, modulate DAT surface localization, and modulate DA uptake and efflux via DAT. We demonstrate that each agonist exhibits unique pharmacology with respect to effects on DAT. These clinically relevant findings may inform future efforts to identify optimal TAAR1 agonists most likely to translate as effective therapeutics for psychiatric disorders.

## 2 Materials and methods

### 2.1 Drugs and reagents

[^125^I]methyl (1R,2S,3S)-3-(4-iodophenyl)-8-methyl-8-azabicyclo[3.2.1]octane-2-carboxylate ([^125^I]RTI-55) and [^3^H]DA were purchased from PerkinElmer Revvity Life and Analytical Sciences. Cocaine and d-amphetamine were generously supplied by the National Institute on Drug Abuse Drug Supply Program. Mazindol, phorbol 12-myristate 13-acetate (PMA), and RO5166017 were purchased from Cayman Chemical. RO5256390 and ulotaront were purchased from MedChemExpress. Hygromycin B was purchased from GoldBio. Drugs were first dissolved at a concentration of 10 mM in DMSO and then further diluted into culture medium at the concentrations designated. Rabbit anti–RFP primary antibody (600-401-379, 1:2500) was purchased from Rockland Immunochemicals, rabbit anti-Na^+^/K^+^ ATPase primary antibody (AB76020, 1:2000) was purchased from Abcam, and donkey anti–rabbit horseradish peroxidase secondary antibody (1:10,000) was purchased from Jackson ImmunoResearch.

### 2.2 Animals

Male and female C57BL/6 mice (10–20 weeks old) were used in this study. Mice were group-housed in filtered polycarbonate cages (28 cm × 18 cm × 13 cm) on ECO-Fresh bedding (Absorption Corporation) with ad libitum access to water and rodent chow (5LOD, 5.0% fat content; Purina Mills). Housing was maintained at 21 ± 1 °C with a 12-hour light/dark schedule. All procedures were conducted in accordance with the National Institutes of Health Guide for the Care and Use of Laboratory Animals and with approval by the Veterans Affairs Portland Health Care System Institutional Animal Care and Use Committee.

### 2.3 Cell lines

All cell-based studies were performed in HEK293T (CRL-3216) or *TAAR1* KO HEK293 cells (a gift from the Amara laboratory, National Institute on Mental Health). HEK293/293T cells endogenously express low levels of TAAR1. Endogenous TAAR1 expression in these lines is insufficient for the levels necessary to conduct pharmacological assays, necessitating the use of TAAR1 overexpression systems for this purpose. Nevertheless, endogenous TAAR1 expression is a confounding factor in studying the role of TAAR1 in HEK293-based systems. To investigate the influence of TAAR1 on DAT, we developed 2 stable cell lines stably expressing the same mScarlet-I-DAT expression construct: one in HEK293T cells that also stably express a TAAR1 construct (“+TAAR1 DAT”), and one in previously developed *TAAR1* KO HEK293 cells engineered using CRISPR to introduce an early stop codon (p.Y27X) into the TAAR1 gene (“TAAR1^−/−^ DAT”). Further description of stable cell line generation is below. Cells were cultured in Dulbecco’s modified Eagle medium (Gibco, catalog no. 12-800-017) supplemented with 10% FetalClone serum (Cytiva; catalog no. SH30080.03) at 37 °C in 5% CO_2_.

### 2.4 Genetic construction of mScarlet-DAT expression plasmid

Expression plasmids containing the full-length coding region of codon-optimized human DAT cDNA with an N-terminal EYFP tag (EYFP-DAT, depositing laboratory: Jonathan Javitch, Addgene #19991) and mScarlet-I cDNA (depositing laboratory: Dorus Gadella, Addgene #137805) were obtained from Addgene.^25^^,^^26^ Expression plasmids were transformed into chemically competent *E. coli* (One Shot TOP10; Thermo Fisher Scientific) according to manufacturer protocol and selected in 100 *μ*g/mL ampicillin (Sigma) and 50 *μ*g/mL kanamycin (Sigma), respectively. Plasmid DNA was prepared using the QIAprep Spin Miniprep Kit (Qiagen) after transformation. The mScarlet-I gene was amplified by polymerase chain reaction (PCR; cloning primers – forward: TATATAATTTAAATGCCACCATGGTGAGCAAGGGCGAG; reverse: TATATATGTACAGCTCGTCCATGCCGCCGG). The EFYP-DAT construct and PCR products were digested sequentially with SwaI (New England Biolabs) and BsrGI-HF (New England Biolabs) at 25 °C for 45 minutes and 37 °C for 45 minutes, respectively, to excise EYFP from the destination vector and prepare the mScarlet-I amplicon for ligation. Digested vectors and inserts were purified on a 1% agarose gel in Tris-acetate-EDTA buffer (40 mM Tris base, 20 mM acetic acid, 1 mM EDTA, pH 8.0) and extracted using the QIAquick Gel Extraction Kit (Qiagen). Ligation reactions were prepared using T4 DNA Ligase (New England Biolabs) according to manufacturer protocol, and ligation was performed overnight at 16 °C to generate the mScarlet-I-DAT expression construct. Ligation products were then transformed into chemically competent *E. coli* (One Shot TOP10) according to manufacturer protocol. Transformed cells were plated on Luria-Bertani agar plates supplemented with ampicillin (Sigma) and incubated overnight at 37 °C. Colonies were screened by PCR, and positive results were confirmed with Sanger sequencing (sequencing primers – forward: GTCGACCGCAAGTTGGACATC; reverse: GCAGGAAGTCGATTTTCTTGCC).

### 2.5 Generation of stable cell lines

The subcloned mScarlet-I-DAT expression construct was transfected into HEK293T cells stably expressing human TAAR1 with a C-terminal mGFP tag (previously developed^27^) or *TAAR1* KO HEK293 cells (previously developed^8^) by polyethylenimine (PEI)–mediated transfection (1 *μ*g/mL; PEI:DNA = 1:2). Cells were selected in media containing 50 *μ*g/mL hygromycin B (Sigma) and 2 *μ*g/mL puromycin (Sigma).

### 2.6 Radioligand binding assays

Affinities of test compounds at DAT were determined by [^125^I]RTI-55 radioligand binding experiments performed as previously described.^28^ Briefly, *TAAR1* KO HEK293 cells stably expressing human DAT were rinsed with Ca2^+^-/Mg^2+^-free PBS and lysed in lysis buffer (2 mM HEPES, 1 mM EDTA). Cell lysates were pelleted by centrifugation at 30,000*g* for 20 minutes and resuspended and homogenized in 0.32 M sucrose. Membrane aliquots (10–30 *μ*g of protein per assay) were incubated with test compounds (indicated concentrations) and [^125^I]RTI-55 (40 pM) in duplicate in Krebs–HEPES buffer (25 mM HEPES, 122 mM NaCl, 5 mM KCl, 1.2 mM MgSO_4_, 2.5 mM CaCl_2_, 1 *μ*M pargyline, 100 *μ*M tropolone, 2 mg glucose/mL, 1 mM ascorbic acid, pH 7.4) in a final volume of 250 *μ*L for 90 minutes at room temperature in the dark. Nonspecific binding was determined in the presence of 10 *μ*M mazindol. Bound radioligand was separated from free radioligand by filtration through PerkinElmer Filtermat A filters using a 96-well Tomtec cell harvester. After drying, scintillation fluid was added to the filters, and radioactivity was determined using a PerkinElmer 1205 Betaplate scintillation counter.

### 2.7 Transport inhibition assays

Potencies of test compounds to inhibit DA uptake by DAT were determined using [^3^H]DA uptake competition assays performed as previously described.^29^ Briefly, *TAAR1* KO HEK293 cells stably expressing human DAT were removed from plates by gentle scraping and trituration in Krebs–HEPES buffer. Cells and test compounds (indicated concentrations) were added to vials and preincubated for 10 minutes at 25 °C before initiating [^3^H]DA uptake (20 nM final concentration, final volume = 500 *μ*L). Nonspecific uptake was determined in the presence of 5 *μ*M mazindol. [^3^H]DA uptake was conducted for 10 minutes and terminated by filtration as described above through Filtermat A filters presoaked in 0.05% PEI. Leftover cell suspension was homogenized and used to determine protein concentrations with the BCA protein assay kit (Pierce).

### 2.8 Transporter saturation assays

Heterologous effects of test compounds on DA transport were determined by [^3^H]DA saturation uptake assays. Briefly, HEK293T cells stably expressing human TAAR1 and human DAT (or *TAAR1* KO HEK293 cells stably expressing human DAT) were pretreated with test compounds in culture media in situ for 40 minutes at 37 °C. Pretreatment media (containing test compounds) was removed by aspiration, and cells were rinsed twice with Ca^2+^-/Mg^2+^-free PBS to remove residual test compounds. Nonspecific uptake was determined in the presence of 5 *μ*M mazindol. [^3^H]DA uptake was conducted for 10 minutes and terminated by filtration as described above.

### 2.9 Transporter saturation assays in synaptosomes

Synaptosomes were isolated as previously described.^7^ Briefly, striatal tissue from untreated mice was homogenized in Neurobasal Medium (Thermo Fisher Scientific) supplemented with 10% heat-inactivated HyClone FetalClone serum. Striatal homogenates from C57BL/6J mice were preincubated with vehicle or drug for 40 minutes at 37 °C in 5% CO_2_ and then placed on ice to restrict further trafficking or endocytosis during additional processing to generate synaptosomes. First, sucrose was added (final concentration = 0.32M) and homogenates were centrifuged (800*g*, 4 °C, 10 minutes). The supernatant (S1) was collected and then centrifuged (10,000*g*, 4 °C, 20 minutes). The resulting pellets (P2), which are isolated synaptosomes, were resuspended in Krebs–HEPES buffer with 1 *μ*M butaclamol to proceed to saturation uptake assays, which were conducted as described above after incubation with [^3^H]DA for 10 minutes.

### 2.10 DA release assays

AMPH-induced release of preloaded [^3^H]DA from HEK293T cells stably expressing human TAAR1 and human DAT (or *TAAR1* KO HEK293 cells stably expressing human DAT) was measured as previously described.^30^^,^^31^ Briefly, confluent cells cultured in 15-cm tissue culture dishes were scraped into 8 mL of Krebs–HEPES buffer and centrifuged at 500*g* for 7 minutes. To load cells with [^3^H]DA, pellets were resuspended in buffer and incubated with [^3^H]DA (120 nM) for 15 minutes at 30 °C. Cells were then centrifuged (500*g*, 7 minutes), resuspended in buffer, and loaded in each channel of a 20-channel Suprafusion 2500 device (Brandel) using polyethylene discs (Brandel) in the reaction tubes. Perfusion of buffer (0.8 mL/min) was conducted for 30 minutes, with the last 6 minutes (3 × 2-minute fractions) collected. Cells were then perfused with AMPH solutions beginning at *t* = 30 minutes, with 22 minutes (11 × 2-minute fractions) of effluent collected. At *t* = 52 min, 1% SDS was perfused to quantify the remaining radioactivity in the cells (10 minutes, 4 × 2.5-minute fractions of effluent collected). Released [^3^H]DA in each fraction was determined using conventional liquid scintillation counting to quantify radioactivity.

### 2.11 Biotinylation of membrane proteins and western blotting

Cells were grown to confluence in 6-well tissue culture plates. To quantify DAT cell surface localization following treatment, cells were treated with drugs or vehicle in culture medium at 37 °C for 40 minutes, media was removed, and culture plates were placed on ice. Cells were then incubated with 1 mg/mL NHS-SS-biotin (Pierce) in PBS at 4 °C with gentle agitation for 60 minutes. The biotinylation reaction was quenched by rinsing with 0.1 M glycine in PBS. Cells were then lysed in radioimmunoprecipitation assay lysis buffer (Millipore) with protease inhibitor (Roche) for 30 minutes at 4 °C. Samples were centrifuged at 13,300*g*, and the supernatants were reserved. The protein concentration of each sample was determined using the BCA protein assay kit (Pierce). Equal protein amounts from each sample were incubated with immobilized deglycosylated avidin (NeutrAvidin; Pierce) for 60 minutes at 25 °C with end-over-end rotation to isolate biotinylated proteins. Proteins were eluted from avidin beads in 50 *μ*L of Laemmli sample buffer (Sigma) with 5% *β*-mercaptoethanol for 10 minutes at 65 °C. Samples were separated by SDS-PAGE and transferred to polyvinylidene difluoride membranes for western blotting and detection. Membranes were blocked with 5% nonfat dry milk in Tris-buffered saline (20 mM Tris, 150 mM NaCl) with 0.05% Tween 20 at room temperature for 30 minutes and then subsequently incubated at 4 °C overnight with primary antibody (anti–RFP transporter antibody to detect mScarlet-I-DAT; anti–Na^+^/K^+^ ATPase loading control). The immunoblots were incubated for 60 minutes at room temperature with horseradish peroxidase–conjugated donkey anti–rabbit secondary antibody (1:10,000), and immune complexes were visualized by chemiluminescence.

### 2.12 Data analysis

Competition and saturation binding and uptake data were analyzed by nonlinear regression using GraphPad Prism 10.0, with IC_50_ values converted to *K*_i_ values using the Cheng–Prusoff correction.^32^ For release assays, fractional release was defined as the amount of radioactivity in a fraction divided by the total radioactivity remaining in the sample, which was determined by summing the counts per minute in that fraction and all later fractions. For each time course, the area under the curve (AUC) was calculated using GraphPad Prism 10.0 with the baseline defined as the average of the 2 lowest fractions of that time course. For each experiment, the AUC of basal release in the absence of RO5166017 or AMPH was subtracted from each AUC before normalizing to the percentage of maximal AMPH-stimulated release.^10^

Immunoblots were analyzed by densitometry using Fiji^33^ and were normalized to Na^+^/K^+^ ATPase within each experiment as an internal control before normalizing to vehicle to facilitate comparison between treatment groups. Treatments for which transfer was poor were excluded from the analysis. For all experiments, data are presented as mean ± SD, and in some cases with confidence intervals (CIs). Statistical analyses (described below) were performed using GraphPad Prism 10.0. For most statistical comparisons, we performed one-way (usually when determining whether different drugs had differing effects) or two-way ANOVAs (to determine whether differing drug effects interacted with TAAR1 genotype) depending on the structure of the experiment. When standard deviation clearly differed between experimental groups, we transformed the data to equalize variance before proceeding to one-way or two-way ANOVA to determine significance. When we were interested in a comparison between 2 groups with unequal variance for which transformation was not possible, we used a Welch’s *t* test (unequal variances *t* test). For post hoc testing, we performed either Dunnett’s multiple comparisons test (typically after one-way ANOVA to compare drug-treated to vehicle-treated groups) or Tukey’s multiple comparisons test (typically after two-way ANOVAs, which usually involved both comparisons to vehicle-treated groups and same drug or vehicle-treated groups across genotypes). Values of *P* < .05 were considered significant for all tests. All instances in which we use the word significant denote statistical significance.

## 3 Results

### 3.1 TAAR1 agonists RO5166017 and RO5256390 directly interact with DAT to inhibit DA uptake

**Fig. 1:**
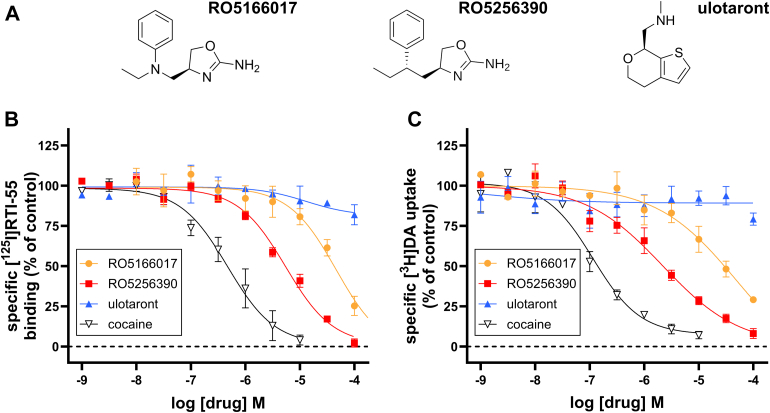
TAAR1 agonists RO5166017 and RO5256390 directly interact with DAT. (A) Structures of TAAR1 agonists RO5166017, RO5256390, and ulotaront (SEP-363856). (B) Competition binding curves of TAAR1 agonists and cocaine in [^125^I]RTI-55 radioligand binding assays at DAT. Plotted values are mean ± SD from 4 independent experiments, with duplicate measurements at each concentration of drug. (C) Inhibition of DA uptake by TAAR1 agonists and cocaine in [^3^H]DA uptake assays at DAT. Plotted values are mean ± SD from 6 independent experiments, with duplicate measurements at each concentration of drug. Fitted results and statistics summarized in Table 1.

Previous characterization of the TAAR1 agonists RO5166017, RO5256390, and ulotaront (Fig. 1A) included broad selectivity screens consisting of receptor binding and enzyme assays. In those selectivity screens, a fixed concentration (10 *μ*M) of drug was evaluated. Inhibition of human DAT [^3^H]BTCP (1-[1-(2-Benzo[b]thiopheneyl)cyclohexyl]piperidine hydrochloride) binding was reported for RO5166017 and RO5256390 (64.0% and 94.1% inhibition of control binding, respectively).^18^^,^^19^ Ulotaront was similarly evaluated, although results from those screens were not published.^34^ Thus, we sought to extensively characterize the interactions of these compounds with DAT by conducting [^125^I]RTI-55 radioligand binding experiments to test the ability of each TAAR1 agonist to bind to the cocaine/RTI-55 binding site and used [^3^H]DA uptake experiments to assess direct inhibition of DA uptake in HEK293 cells stably expressing human DAT and no TAAR1. Binding affinities and potencies for TAAR1 agonists RO5166017, RO5256390, and ulotaront and the DAT inhibitor, cocaine, were assessed. RO5166017 and RO5256390 both bound DAT with similar (1–10 *μ*M) affinities (see Fig. 1B for binding curves and Table 1 for summary of results), although both compounds had lower affinity for DAT than the submicromolar affinity of cocaine (one-way ANOVA after converting to p*K*_i_ to equalize variances, *F*(2,9) = 101.7, *P* < .0001; Tukey’s multiple comparisons test, RO5166017 versus cocaine, adjusted *P* < .0001; RO5256390 versus cocaine, adjusted *P* < .0001). Ulotaront did not displace [^125^I]RTI-55 binding at any concentration tested, demonstrating that it does not directly bind to DAT at the cocaine/RTI-55 binding site. In [^3^H]DA competition uptake experiments, the same rank order potency was observed as the rank order affinities determined in radioligand binding experiments (Fig. 1C; Table 1). RO5166017 and RO5256390 both directly inhibited DA uptake, albeit with lower potency than cocaine (one-way ANOVA after converting to pIC_50_s to equalize variances, *F*(2,15) = 95.59, *P* < .0001; Tukey’s multiple comparisons test, RO5166017 versus cocaine, adjusted *P* < .0001; RO5256390 versus cocaine, adjusted *P* < .0001). Ulotaront, which did not bind DAT appreciably in the radioligand binding experiments, had no effect on DA uptake in these experiments.

**Table 1:** Affinity and potency of TAAR1 agonists and standard compound (cocaine) at the human DAT Data were normalized to specific binding or specific uptake in the absence of drugs. Drugs were tested in binding and uptake assays at concentrations ranging from 1 nM to 100 *μ*M. Cocaine was used as a standard compound that binds to DAT and inhibits DA uptake. RO5166017 and RO5253690 also bound to and inhibited DAT uptake of DA, whereas ulotaront had no effect in either assay. One-way ANOVA (p*K*_i_), *F*(2,9) = 101.7, *P* < .0001; Tukey’s multiple comparisons test, RO5166017 versus cocaine, adjusted *P* < .0001; RO5256390 versus cocaine, adjusted *P* < .0001). One-way ANOVA (pIC_50_), *F*(2,15) = 95.59, *P* < .0001; Tukey’s multiple comparisons test, RO5166017 versus cocaine, adjusted *P* < .0001; RO5256390 versus cocaine, adjusted *P* < .0001).

Drug	Inhibition of [^125^I]RTI-55 Binding p*K*_i_ ± SD (*n*)	CI		Inhibition of [^3^H]DA Uptake pIC_50_ ± SD (*n*)	CI	
RO5166017	4.66 ± 0.16 (4)	4.5	4.82	4.76 ± 0.36 (6)	4.48	5.05
RO5256390	5.50 ± 0.25 (4)	5.25	5.75	5.73 ± 0.23 (6)	5.55	5.92
Ulotaront	<4 (4)	n/a	n/a	<4 (6)	n/a	n/a
Cocaine	6.47 ± 0.10 (4)	6.39	6.56	6.80 ± 0.13 (6)	6.70	6.90

n/a, not applicable.

### 3.2 Pretreatment with different TAAR1 agonists differentially affects DA uptake at DAT

**Fig. 2:**
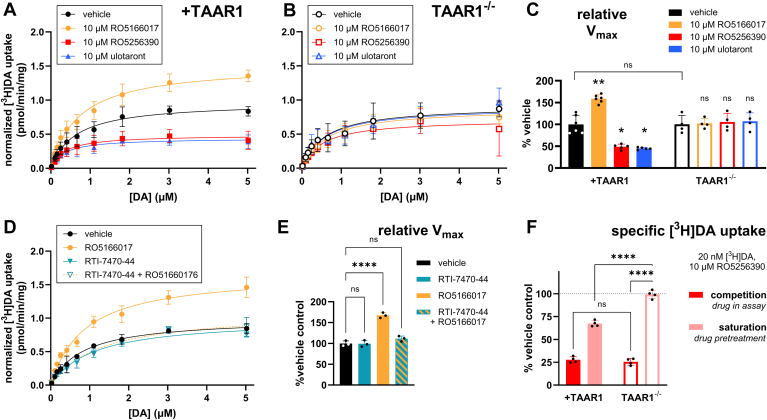
TAAR1 agonists differentially affect DA uptake at DAT via TAAR1-dependent heterologous mechanisms. (A) In HEK293T cells stably expressing TAAR1 and DAT (+TAAR1), pretreatment with RO5166017 significantly increased DA uptake (*V*_max_) relative to vehicle, while treatment with RO5256390 and ulotaront significantly decreased *V*_max_ (see Table 2 for relevant fitted parameters and associated statistical summary corresponding to panels 2A–2C). Drug treatment did not significantly affect transporter affinity for substrate (*K*_m_; see Table 2). (B) In *TAAR1* KO HEK293 cells stably expressing DAT (TAAR1^−/−^), there was no effect of RO5166017, RO5256390, or ulotaront on *V*_max_ or *K*_m_. (C) Summary of relative maximal uptake (normalized *V*_max_) values obtained from fitting data in panels 2A and 2B, illustrating changes in *V*_max_ in +TAAR1 cells, ∗*P* < .05 and ∗∗*P* < .01. *P* values reflect statistical analyses performed on raw *V*_max_ values (statistics summarized in detail in Table 2); normalized data is presented for illustrative purposes. *V*_max_ values for each treatment condition were normalized to vehicle *V*_max_ by experiment and genotype. Vehicle *V*_max_ values are normalized to the mean vehicle *V*_max_ obtained from all experiments conducted in +TAAR1 cells. Data represent mean ± SD from 4–6 independent experiments performed in triplicate. (D) Pretreatment with the TAAR1 antagonist RTI-7470-44, which had no effect on *V*_max_, blocked the effect of RO5166017 on DA uptake (see Table 3 for summary of values and ANOVA statistics). (E) *V*_max_ summary corresponding to panel D and illustrating significant effects (adjusted ∗∗∗∗*P* < .0001). (F) Relative specific [^3^H]DA uptake (pmol/min/mg) values from competition uptake experiments (Fig. 1B) and saturation uptake experiments (panels A and B from this figure) where total [^3^H]DA concentration = 20 nM and RO5256390 = 10 *μ*M. In direct competition uptake experiments, 10 *μ*M RO5256390 inhibited [^3^H]DA uptake by roughly 75% in both +TAAR1 and TAAR1^−/−^ cells (see Table 4 for detailed summary of values and statistics). In saturation uptake assays, pretreatment with 10 *μ*M RO5256390 inhibited [^3^H]DA uptake in +TAAR1 cells but not TAAR1^−/−^ cells (adjusted ∗∗∗∗*P* < .0001). In all experiments, values were normalized to the amount of protein in each sample. Measurements of uptake were conducted in triplicate, and data points and means of those triplicates are plotted ± SD. Data presented in panels 2A–2C and 2F were analyzed by two-way ANOVA with genotype and drug treatment as factors with Tukey’s multiple comparisons test. Complete ANOVA statistics are provided in Table 2 (2A–2C), Table 3 (2D–2E), and Table 4 (2F). Data shown in panels 2D and 2E were analyzed by ordinary one-way ANOVAs with Dunnett’s multiple comparisons test.

AMPH, a nonselective TAAR1 agonist, reduces DA uptake, in part through TAAR1-mediated DAT internalization.^8^ To determine if other TAAR1 agonists have similar effects on DA uptake, we conducted [^3^H]DA saturation uptake experiments to determine the effects of TAAR1 agonist *pretreatment* on DA uptake. In the direct conditions, TAAR1 agonist was coincubated with [^3^H]DA. In contrast, pretreatment conditions were designed so that TAAR1 agonists were not present during the uptake assays, including a washout step that preceded measurement of uptake, avoiding direct inhibition of DA uptake/DAT binding. Data were fit to yield estimates of the maximal uptake (*V*_max_) and of affinity of DAT for DA (*K*_m_). In cells stably expressing TAAR1 and DAT, RO5256390 and ulotaront pretreatment both resulted in a reduced *V*_max_ relative to vehicle without altering *K*_m_ (Fig. 2, A and C; Table 2; also see Fig. 2 and Table 2 for detailed results and two-way ANOVA and post hoc statistics summary). Intriguingly, RO5166017 pretreatment increased DA uptake compared to vehicle without altering the *K*_m_ value (Fig. 2A; Table 2).

**Table 2:** Effect of TAAR1 agonist pretreatment on human DAT maximum velocity of substrate transport (*V*_max_) and affinity for substrate (*K*_m_) Data were analyzed by two-way ANOVA with Tukey’s multiple comparisons test; adjusted *P* values and significant ANOVA results are reported; ∗ *P* < .05; ∗∗ *P* < .01; ∗∗∗*P* < .0001; ns, not significant. All 3 drugs modulated *V*_max_ but not *K*_m_ in a TAAR1-dependent manner (significant main effect of drug and genotype factors with a significant interaction).

*+TAAR1*	*V*_max_ ± SD (n)	CI			Adjusted *P* value	*K*_m_ ± SD (n)	CI	
Treatment	*(pmol/min/mg)*				*(Compared to +TAAR1 Vehicle)*	*(nM)*		
Vehicle	1.59 ± 0.34 (6)	1.45	1.73		--	746 ± 320 (6)	615	877
10 *μ*M RO5166017	2.50 ± 0.42 (6)	2.33	2.67	∗∗	.0093	823 ± 290 (6)	705	941
10 *μ*M RO5256390	0.78 ± 0.20 (5)	0.69	0.87	∗	.0334	770 ± 720 (5)	448	1092
10 *μ*M ulotaront	0.71 ± 0.09 (5)	0.67	0.75	∗	.0194	590 ± 760 (5)	250	930

*TAAR1*^*−/−*^	*V*_max_ ± SD (*n*)	CI			Adjusted *P* Value	*K*_m_ ± SD (*n*)	CI	
Treatment	*(pmol/min/mg)*				*(Compared to TAAR1*^*−/−*^*Vehicle)*	*(nM)*		
Vehicle	1.63 ± 0.34 (4)	1.46	1.8		--	631 ± 260 (4)	501	761
10 *μ*M RO5166017	1.67 ± 0.84 (4)	1.25	2.09	ns	.9996	690 ± 260 (4)	560	820
10 *μ*M RO5256390	1.80 ± 0.92 (4)	1.34	2.26	ns	.9572	718 ± 180 (4)	628	808
10 *μ*M ulotaront	1.77 ± 0.70 (4)	1.46	2.08	ns	.9741	818 ± 580 (4)	529	1109

	Adjusted *P* Value
*+TAAR1* vs *TAAR1^−/−^*	*(V*_*max*_*)*
Vehicle	.8913, ns
10 *μ*M RO5166017	.0087, ∗∗
10 *μ*M RO5256390	.0026, ∗∗
10 *μ*M ulotaront	.0019, ∗∗
Two-way ANOVA: *V*_max_	
Factor 1: genotype	*F*(1,30) = 4.39, *P* = .0448, ∗
Factor 2: drug	*F*(3,30) = 6.57, *P* = .0015, ∗∗
Interaction	*F*(3,30) = 8.86, *P* = .0002, ∗∗∗
Two-way ANOVA: *K*_m_	
Factor 1: genotype	F(1,30) = 0.01, *P* = .9099, ns
Factor 2: drug	F(3,30) = 0.04, *P* = .9883, ns
Interaction	F(3,30) = 0.28, *P* = .8412, ns

To evaluate the role of TAAR1 in these effects, we conducted the same experiments in TAAR1^−/−^ cells stably expressing DAT (HEK293 cells endogenously express low levels of TAAR1) and for RO5166017 in the presence of the TAAR1 antagonist RTI-7470-44. There were no significant effects of drug treatment on *V*_max_ or *K*_m_ values in TAAR1^−/−^ cells (Fig. 2C; see Table 2 for results and two-way ANOVA and post hoc statistics summary) or in the presence of RTI-7470-44 (Fig. 2, D and E; Table 3).

**Table 3:** RT-7470-44 blocks the ability of RO5166017 pretreatment to enhance DA uptake in +TAAR1/DAT cells Data were analyzed by one-way ANOVA (main factor: treatment) with post hoc Dunnett’s multiple comparisons test; ∗ *P* < .05; ∗∗ *P* < .01; ∗∗∗*P* < .0001; ns, not significant. 1 *μ*M RTI-7470-44 alone has no effect on *V*_max_ but blocks the effect of 10 *μ*M RO5166017.

*+TAAR1*								
Treatment	*V*_max_ ± SD (*n*)	CI		Adjusted *P* Value		*K*_m_ ± SD (*n*)	CI	
	*(pmol/min/mg)*			*(Compared to Vehicle)*		*(nM)*		
Vehicle	1.63 ± 0.11 (3)	1.57	1.69	--		839 ± 160 (3)	747	931
1 *μ*M RTI-7470-44	1.62 ± 0.12 (3)	1.55	1.69	0.9997	ns	882 ± 92 (3)	829	935
10 *μ*M RO5166017	2.73 ± 0.17 (3)	2.63	2.83	<0.0001	∗∗∗∗	1120 ± 210 (3)	999	1241
1 *μ*M RTI-7470-44 + 10 *μ*M RO5166017	1.81 ± 0.17 (3)	1.71	1.91	0.4710	ns	1150 ± 230 (3)	1017	1283
One-way ANOVA (*V*_max_)	*F*(3,8) = 36.7, *P* < .0001							
One-way ANOVA (*K*_m_)	*F*(3,8) = 3.4, *P* = .0761							

To confirm effective washout of TAAR1 agonist in these experiments, we compared the effect of the TAAR1 agonists under identical uptake conditions (10-minute coincubation with 20 nM [^3^H]DA) with and without washout, comparing direct, short coincubation with TAAR1 agonist (10 minutes) versus longer pretreatment (40 minutes) with TAAR1 agonist followed by washout. We found that there was no decrease in uptake under pretreatment conditions in TAAR1^−/−^ cells, ruling out a direct effect on uptake that we know from the direct experiment in Fig. 1 is inhibitory (Fig. 2F; see competitive “direct” conditions versus “saturation” heterologous conditions). Furthermore, the level of inhibition under competition conditions was the same in +TAAR1 cells as it was in TAAR1^−/−^, which further supports no measurable TAAR1-mediated effect in the direct assay conditions (see Table 4 for one-way ANOVA statistics and post hoc testing).

**Table 4:** Inhibition of uptake by RO5256390 in saturation assays testing drug pretreatment effects depends on TAAR1 whereas competitive inhibition does not Data were analyzed by one-way ANOVA with post hoc Dunnett’s multiple comparisons tests, and adjusted *P* values and significant ANOVA results are reported. ∗ *P* < .05; ∗∗ *P* < .01; ∗∗∗*P* < .0001; ns, not significant. The pattern of inhibition of uptake under the 2 sets of conditions suggests that they adequately separate direct (which do not depend on TAAR1) from TAAR1-dependent (heterologous) effects (which are not detectable when using brief incubation “direct” conditions).

	Specific [^3^H]DA Uptake					
Assay	Mean ± SD (*n*)	CI		Mean ± SD (*n*)	CI	
	(% of Vehicle Control)			(% of Vehicle Control)		
	+TAAR1			TAAR1^−/−^		
Competition uptake	27.7 ± 3.4 (4)	26	29.4	25.5 ± 3.6 (4)	23.7	27.3
Saturation uptake	67.3 ± 3.4 (4)	65.6	69	99.2 ± 4.4 (4)	97	101.4
Two-way ANOVA						
Factor 1: genotype	F(1,12) = 63.4, *P* < .0001 ∗∗∗∗					
Factor 2: assay	F(1,12) = 923, *P* < .0001 ∗∗∗∗					
Interaction	F(1,12) = 84.1, *P* < .0001 ∗∗∗∗					
Tukey’s multiple comparisons test	Adjusted *P* value					
+TAAR1 (competition) vs +TAAR1 (saturation)	<.0001, ∗∗∗∗					
TAAR1^−/−^ (competition) vs TAAR1^−/−^ (saturation)	<.0001, ∗∗∗∗					
+TAAR1 (competition) vs TAAR1^−/−^ (competition)	.8275, ns					
+TAAR1 (saturation) vs TAAR1^-/-^ (saturation)	<.0001, ∗∗∗∗					

We finally assessed the time course of the effect of RO5166017 in +TAAR1 cells. A one-way ANOVA with RO5166017 pretreatment time as the main factor was significant, *F*(5,12) = 19.29, *P* < .0001; *n* = 3 for each treatment. Post hoc testing (Dunnett’s multiple comparisons test) found no change in *V*_max_ relative to 15-minute vehicle pretreatment (1.61 ± 0.11, CI = 1.48–1.73) after pretreatment with RO5166017 for 10 minutes (1.53 ± 0.19, CI = 1.31–1.74, adjusted *P* = .9677) or 15 minutes (1.64 ± 0.13, CI = 1.49–1.78, adjusted *P* = .9977), in contrast to the increase seen at 30 minutes (2.12 ± 0.09, CI = 2.03–2.22, adjusted *P* = .0122), 40 minutes (2.44 ± 0.12, CI = 2.30 – 2.58, adjusted *P* = .0003), and 60 minutes (2.47 ± 0.30, CI = 2.14–2.81, adjusted *P* = .0002).

### 3.3 Pretreatment with low concentrations of RO5166017 potentiates AMPH-induced DA efflux

**Fig. 3:**
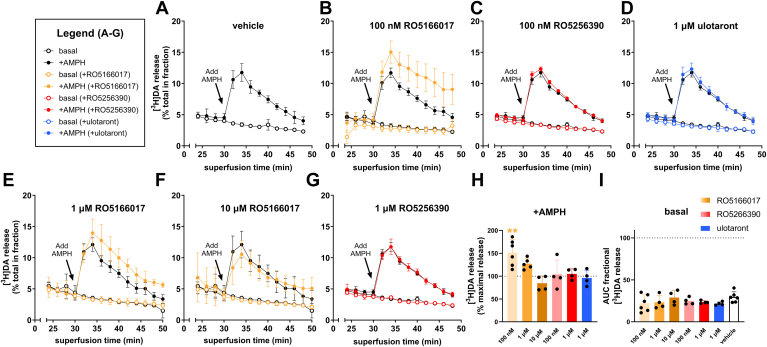
RO5166017 modulates AMPH-induced [^3^H]DA release. Time courses of average fractional [^3^H]DA release induced by AMPH were conducted after 30 minute preincubation with varying concentrations of TAAR1 agonist. AMPH-induced release after lower concentrations of TAAR1 agonist (<IC_50_ for direct inhibition of DA uptake at DAT for RO5166017 and R5256390) revealed that, compared to (A) vehicle pretreatment, (B) 100 nM RO5166017 decreased DA release, while (C) 100 nM RO5256390 and (D) 1 *μ*M ulotaront had no effect. RO5166017 and RO5256390, which directly bind to and inhibit DA uptake via DAT at higher (micromolar) concentrations, had no effect on DA release at (E) 1 *μ*M and (F) 10 *μ*M RO5166017 and (G) 1 *μ*M RO5256390. (H) The AUC for AMPH-induced DA release for concentration of TAAR1 agonist tested was normalized to vehicle and compared using a one-way ANOVA, which indicated a significant effect of treatment, *F*(6,24) = 6.304, *P* = .0004. Post hoc testing with Dunnett’s multiple comparison test indicated a significant increase in DA release after 100 nM RO5166017 (*P* = .004, *n* = 6) but no other treatment (1 *μ*M RO5166017, *P* = .2527, *n* = 5; 10 *μ*M RO5166017, *P* = .6914, *n* = 4; 100 nM RO5256390, *P* > .9999, *n* = 4; 1 *μ*M RO5256390, *P* = .9998, *n* = 4; 1 *μ*M ulotaront, *P* = .9979, *n* = 4). In panel H, ∗∗*P* < .01 compared with vehicle release. (I) One-way ANOVA to compare effects of treatments versus vehicle on basal AUC, representing basal DA release in response to TAAR1 agonist alone, indicated that none of the TAAR1 agonists affected DA release, *F*(6,25) = 1.031, *P* = .4290.

AMPH induces nonvesicular release of DA by promoting DA reverse transport/release (efflux) through DAT.^10^^,^^35^ To further characterize the effects of RO5166017, RO5256390, and ulotaront on DAT, we next assessed their effects on AMPH-induced efflux. Given the unique effect of RO5166017 pretreatment on uptake, we suspected that RO5166017 might also exert unique heterologous effects on AMPH-induced efflux. To measure DA efflux, we performed superfusion assays to quantify [^3^H]DA release in cells stably expressing TAAR1 and DAT versus TAAR1^−/−^ cells stably expressing DAT. As an initial step, we used low concentrations of RO5166017 and RO5256390 (well below concentrations sufficient to exert substantial direct effects on DAT) to better assess their potential heterologous effects, and a high concentration of ulotaront, which does not bind to DAT and which we expected would not exert any direct or heterologous effect. We measured DA efflux in the presence and absence of AMPH. We found that, unlike vehicle (Fig. 3, A–G),100 nM RO5166017 potentiated AMPH-induced DA efflux (Fig. 3, B and H, see Fig. 3 caption for one-way ANOVA and post hoc Dunnett’s statistics), while 100 nM RO5256390 and 1 *μ*M ulotaront had no effect (Fig. 3, C, D, and H). To explore whether direct effects of RO5166017 and RO5256390, which could be relevant at higher (micromolar) concentrations at which direct and heterologous effects would co-occur, if present, we examined the effect of 1 and 10 *μ*M RO5166017 and 1 *μ*M RO5256390. While we found no effect of a higher concentration of RO5256390 (Fig. 3, G and H), we found that the potentiating effect of RO5166017 disappeared at the higher concentrations of 1 *μ*M (where there was a consistent nonsignificant trend toward an increase) and 10 *μ*M (Fig. 3, E, F, and H). These results suggest that RO5166017 may have a potentiating effect on AMPH-induced DA efflux at lower concentrations (likely TAAR1-mediated) and an opposing (direct) effect at high concentrations, with the result being no net effect at high concentrations.

**Fig. 4:**
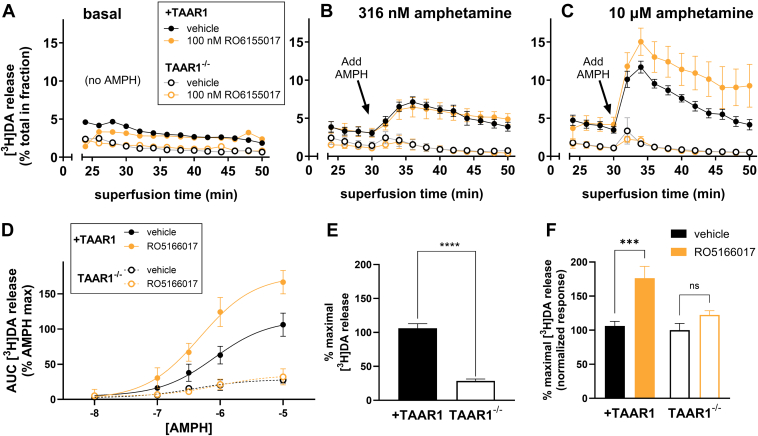
AMPH-stimulated DA release through DAT is potentiated by RO5166017 in a TAAR1-dependent process. The effect of RO5166017 pretreatment on AMPH-stimulated [^3^H]DA release was assessed in +TAAR1 and TAAR1^−/−^ cells after pretreatment with buffer (vehicle) or 100 nM RO5166017. [^3^H]DA release time courses of average fractional release in the presence of (A) vehicle (basal release), (B) 316 nM AMPH, and (C) 10 *μ*M AMPH suggest a potentiation of AMPH-induced DA release at higher concentrations of DA. Data are normalized to percentage release of [^3^H]DA remaining in cells at each time point. The last 3 buffer fractions prior to addition of AMPH and the 11 fractions in the presence of AMPH are shown. (D) Concentration-response curves fit AMPH-stimulated [^3^H]DA release data in +TAAR1 and TAAR1^−/−^ cells following pretreatment with vehicle or 100 nM RO5166017 show a potentiating effect of drug in +TAAR1 cells. The AUC for each AMPH concentration was normalized to the maximal effect of AMPH in vehicle-pretreated cells (eg, in the absence of RO5166017) for that experiment. (E) Efficacy (*E*_max_) of AMPH to release [^3^H]DA from +TAAR1 and TAAR1^−/−^ cells was derived from the curves in panel D. The 2 groups were compared using a Welch’s *t* test, which can accommodate unequal variance between groups. Efficacy of AMPH to release [^3^H]DA was significantly reduced in TAAR1^−/−^ cells (*E*_max_ = 28.5% ± 5.7%, *n* = 4, *P* < .0001, *t* = 10.69, df = 6.607) as compared to +TAAR1 cells (*E*_max_ = 106% ± 6.7%, *n* = 6). (F) Data derived from panels A–D for each AMPH concentration were normalized to the maximal effect of AMPH in vehicle-pretreated cells (eg, in the absence of RO5166017) within genotype to prepare normalized concentration-response curves and generate corresponding *E*_max_ values, which equalized variance across groups. The resulting *E*_max_ values were analyzed by two-way ANOVA with genotype and RO5166017 treatment as factors followed by Tukey’s multiple comparison test (adjusted ∗∗∗∗*P* < .0001). The goal was to determine whether RO5166017 pretreatment affected AMPH-stimulated efflux in TAAR1^−/−^ cells. For this panel, two-way ANOVA indicated main effects of genotype (*F*(1,14) = 8.365, *P* < .0118), drug (*F*(1,14) = 19.78, *P* = .0006), and an interaction (*F*(1,14) = 5.271, *P* = .0376), the latter suggesting TAAR1 dependence of the effect of RO5166017. Relative to within-genotype vehicle, RO5166017 pretreatment significantly potentiated AMPH-induced efflux in cells expressing TAAR1 (RO5166017 *E*_max_ = 176% ± 17%, *n* = 4; versus vehicle *E*_max_ = 106% ± 6.7%, *n* = 6; *P* < .0003). RO5166017 had no effect on AMPH-stimulated release in TAAR1^−/−^ cells as compared to vehicle (RO5166017 *E*_max_ = 122% ± 16%, *n* = 3 versus vehicle *E*_max_ = 108% ± 10%, *n* = 4, *P* = .4122). Data in this figure are presented as mean ± SD (*n* = 4–6).

We next determined whether the effect of RO5166017 was TAAR1-dependent. We generated AMPH-induced DA release dose-response curves with and without 100 nM RO5166017 (low concentration selected to prioritize potential heterologous effects) in TAAR1^+/+^ and TAAR1^−/−^ cells also expressing DAT (see Fig. 4, A–C for basal release and a low and high concentration of AMPH; see Fig. 4D for fitted AMPH dose-response curves from which we generated *E*_max_ estimates). In Fig. 4E, we show AMPH-induced *E*_max_ normalized to the maximal DA release for +TAAR1 vehicle. Due to concern the 2 groups had unequal variance, we performed a Welch’s *t* test to first examine whether there was a difference between AMPH-induced *E*_max_ in the 2 groups. We found that AMPH-induced release was lower in TAAR1^−/−^ cells (see Fig. 4 legend for statistics). To confirm whether the effect of RO5166017 was present in +TAAR1 cells and absent in TAAR1^−/−^ cells, we normalized each RO5166017 group to cells of the same TAAR1 genotype treated with vehicle (to equalize variance across groups) and performed a two-way ANOVA (see Fig. 4 legend for statistical results). Results confirmed the effect of RO5166017 on AMPH-induced DA release (as measured by release *V*_max_) was dependent on TAAR1. Of note, we also found that AMPH did not induce DA efflux in TAAR1^−/−^ cells with lower concentrations of [^3^H]DA. This agrees with previous reports that the largest component of AMPH-induced DA efflux is TAAR1-dependent, whereas TAAR1-independent AMPH-induced efflux is detectable only at micromolar [^3^H]DA concentrations.^6^^,^^36^ Together, these results indicate that among the 3 TAAR1 agonists tested, only RO5166017 appears to exert TAAR1-dependent potentiation of AMPH-induced DA efflux. The TAAR1-mediated enhancement in efflux is particularly interesting given that RO5166017 has no TAAR1-mediated effect on efflux alone and is essentially competing with AMPH at TAAR1 to enhance the TAAR1-mediated increase in AMPH-induced DA efflux.

### 3.4 TAAR1 agonists differentially affect DAT localization at the cell membrane

**Fig. 5:**
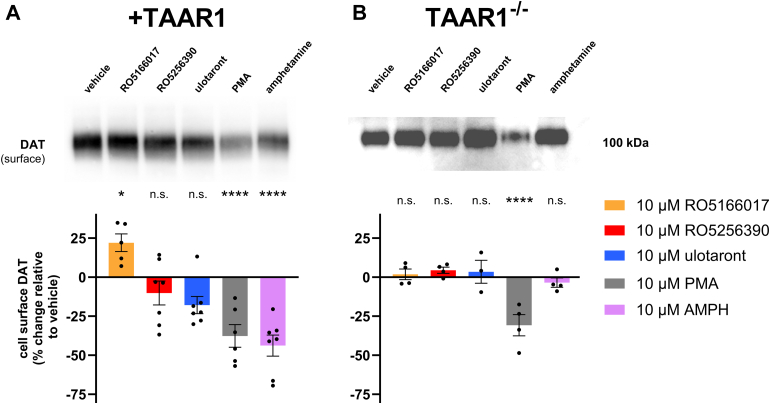
Pretreatment with RO5166017 increases DAT localization at the plasma membrane. Surface biotinylation was conducted following 40-minute pretreatment with TAAR1 agonists (10 *μ*M RO5166017, RO5256390, and ulotaront), vehicle, 10 *μ*M AMPH (which decreases cell surface expression via TAAR1 agonism), or 1 *μ*M PMA (which decreases cell surface expression via a TAAR1-independent, protein kinase C-dependent mechanism). Individual data points for each experiment (*n* = 3–5) are shown with bars representing mean ± SD. Data were analyzed by two-way ANOVA with drug and genotype as factors with Dunnett’s multiple comparisons test for post hoc testing, *F*(1,50) = 8.695, *P* =.0048 for genotype factor; *F*(5,50) = 14.87, *P* = .0001 for treatment factor; *F*(5,50) = 5.512, *P* = .0004 for interaction. (A) In +TAAR1 cells, treatment with RO5166017 increased DAT cell surface localization relative to vehicle treatment (*P =* .0301, *n* = 4), while treatment with PMA and AMPH decreased DAT cell surface localization, as shown previously (*P* < .0001, *n* = 4; *P* < .0001, *n* = 5, respectively). Pretreatment with RO5256390 and ulotaront had no effect (*P* = .4958, *n* = 4; *P* = .0.655, *n* = 4, respectively). (B) The effects of RO5166017 and AMPH on DAT surface localization were absent in TAAR1^−/−^ cells (*P* = .9998, *n* =4; *P* = .9958, *n* = 4, respectively, versus vehicle), while the effect of PMA persisted (*P* = .0094, *n* = 4). RO5256390 and ulotaront also had no effect in TAAR1^−/−^ cells (*P* = .9874, *n* = 4; *P* = .997, *n* = 3, respectively). The representative blot shows total surface biotinylated product for control- and experimental compound-treated cells as assessed by probing for the mScarlet tag. For each experiment, band optical density for each treatment was quantified and normalized to the band optical density for Na^+^/K^+^ ATPase and then expressed as percent change relative to vehicle control to facilitate comparison. Adjusted ∗*P* < .1, ∗∗∗*P* < .001, and ∗∗∗∗*P* < .0001 compared with vehicle.

The observed effects of TAAR1 agonists on DA uptake, as well as the potentiation of AMPH-induced release by RO5166017, suggest that the 3 TAAR1 agonists tested may have different effects on DAT availability at the plasma membrane. In particular, we expected that RO5166017 would increase surface expression of DAT, which would explain both the increase in DA uptake after pretreatment and the potentiation of AMPH-induced DA efflux via DAT. To investigate this possibility, we performed surface biotinylation experiments to determine the effect of pretreatment on DAT surface localization. Cells stably expressing TAAR1 and DAT and TAAR1^−/−^ cells stably expressing DAT were pretreated with TAAR1 agonists for 40 minutes, and surface membrane proteins remaining on the cell surface after treatment were biotinylated. Biotinylated proteins were isolated by affinity purification and separated by SDS-PAGE. AMPH, which induces DAT internalization via a TAAR1-dependent mechanism, and PMA, a protein kinase C activator that induces DAT internalization by a TAAR1-independent mechanism, were used as controls.^8^^,^^37^ The level of cell surface DAT after 40 minutes of RO5166017 treatment was significantly increased relative to vehicle in TAAR1 DAT cells (19% ± 13% increase beyond vehicle-treated samples; see Fig. 5 legend for two-way ANOVA statistics, post hoc adjusted *P =* .0301) (Fig. 5A; see legend for full statistics summary). RO5256390 and ulotaront treatment did not have a significant effect on cell surface localization, and AMPH and PMA treatments decreased cell surface localization (−44% ± 18%, *P* = .0013 and *−*38% ± 18%, *P* < .0001, respectively). The effects of RO5166017 and AMPH were absent in TAAR1 knockout cells, while PMA treatment reduced surface localization in both TAAR1 and TAAR1 knockout cells (Fig. 5B; see legend for statistics summary). These results support the hypothesis that RO5166017 increases DA uptake and potentiates AMPH-stimulated DA release via TAAR1-dependent regulation of DAT surface expression. Interestingly, although the effects of RO5166017 were opposite those of AMPH, which decreased DAT surface localization and DA uptake, both depend on TAAR1.

### 3.5 Pretreatment with different TAAR1 agonists differentially affects DA uptake at DAT in synaptosomes

**Fig. 6:**
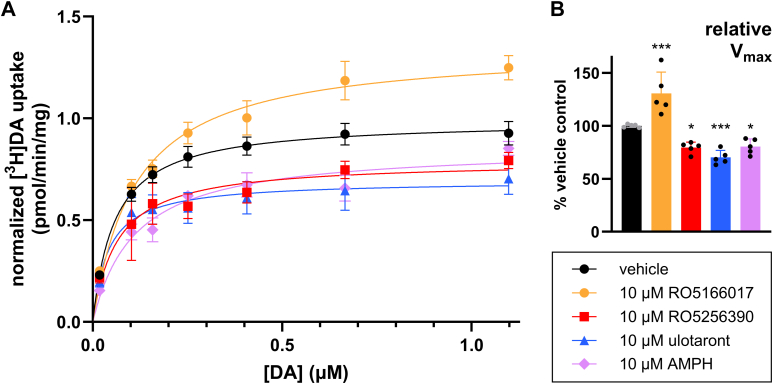
RO5166017 increases whereas RO5256390 and ulotaront decrease DA uptake in mouse striatal synaptosomes. We compared the effects of pretreatment with 4 TAAR1 agonists on DA uptake in mouse striatal synaptosomes. One-way ANOVA (main factor: drug) indicated that the drugs had differing effects on *V*_max_ (*F*(4,20) = 27.42, *P* < .001) but not transporter affinity (*K*_m_) for the DA substrate (*F*(4,20) = 0.7032, *P* = .7032). Dunnett’s multiple comparisons test was used in post hoc testing to determine which drugs had significant effects on *V*_max_ and *K*_m_ relative to vehicle-pretreated control group. (A) Pretreatment with RO5166017 significantly increased DA uptake (*V*_max_ = 1.31 ± 0.20, *P* = .005, *n* = 5) relative to vehicle (*V*_max_ = 1.01 ± 0.1, *n* = 5) while pretreatment with RO5256390, ulotaront, and AMPH significantly decreased dopamine uptake (*V*_max_ = 0.794 ± 0.05, *P* = .0167, *n* = 5; *V*_max_ = 0.702 ± 0.07, *P* = .007, *n* = 5; *V*_max_ = 0.805 ± 0.07, *P* = .0241, *n* = 5, respectively). (B) Summary of normalized *V*_max_ values and *P* values corresponding to panel A. Data represent mean ± SD from 5 independent experiments with measurements performed in duplicate or triplicate. Striatal tissue from 2 mice was pooled for each experiment and aliquoted for drug treatment. For each treatment condition, values were normalized to the amount of protein in each sample, and for each experiment, values were normalized to vehicle *V*_max_ (adjusted ∗*P* < .05 and ∗∗∗*P* < .01, compared to vehicle treatment.)

In cells stably expressing TAAR1 and DAT, RO5166017 pretreatment increased DA uptake while RO5256390 and ulotaront pretreatment reduced uptake relative to vehicle. This left open the question of whether these effects are generalizable to native brain tissue expressing DAT. To examine this question, we prepared synaptosomes from freshly dissected mouse striatal tissue. Using the same pretreatment conditions as in saturation uptake assays conducted in cultured cells, we found that RO5166017 significantly increased DA uptake, while RO5256390 and ulotaront significantly decreased uptake (Fig. 6). These results are consistent with our results from experiments conducted in cultured +TAAR1 DAT cells, suggesting that these differential TAAR1 agonist effects are relevant in native neurons.

## 4 Discussion

TAAR1 agonists represent an emerging class of therapeutics for treatment of disorders characterized by aberrant dopaminergic neurotransmission, but their effects on DAT are not well understood despite the clear clinical relevance of such effects. We discovered that the 3 TAAR1 agonists tested each exhibited unique profiles with respect to DAT function. Many, but not all, effects were TAAR1-dependent. Importantly, while pretreatment of cells with RO526390 and ulotaront decreased DA uptake similarly to AMPH, RO5166017 pretreatment surprisingly increased DA uptake, AMPH-induced DA efflux, and DAT surface localization.

Treatment with AMPH, a nonselective TAAR1 agonist, increased DAT internalization from the plasma membrane, reducing the presence of DAT at the cell surface and decreasing DA uptake in a TAAR1-dependent manner.^8^ In our experiments, we observed these TAAR1-dependent effects of AMPH on surface DAT localization and DA uptake. Whether TAAR1 agonists reduce surface DAT and DA uptake as a general property is a critical question as their promise as therapeutics hinges heavily on their ability to decrease the firing of VTA DA neurons,^18^^,^^22^ although it is worth noting that the psychotogenic AMPHs also reduce VTA DA neuron firing.^38^ Reduced VTA DA neuron firing is generally expected to decrease the amount of DA in the synapse, whereas reduced DA uptake has the opposite effect. Thus, it is necessary to understand the effects of TAAR1 agonists on both release and uptake to discern their net effect on synaptic DA. Importantly, we found that the 3 TAAR1 agonists tested exhibited unique DAT-related pharmacology. Furthermore, none had effects identical to AMPH, although RO5256390 and ulotaront were the most similar in that they reduced DA uptake after pretreatment. Their effect on DA uptake via DAT was dependent on TAAR1, but unlike AMPH, they did not affect surface DAT localization. Intriguingly, RO5166017 increased DA uptake in HEK293T cells overexpressing DAT and TAAR1 and in striatal synaptosomes, which we showed was likely stimulated by the recruitment of DAT to the cell surface via a TAAR1-dependent mechanism. RO5166017, which on its own has no effect on DA efflux through DAT, also potentiated AMPH-induced DA efflux, an effect that was also TAAR1-dependent. In contrast, RO5256390 and ulotaront had no effect on AMPH-induced DA efflux. Together, these results show that although TAAR1 agonists appear to be relatively consistent in their effects on VTA DA neuron firing, they are much less consistent in their effects on DAT surface expression and uptake. This means that some TAAR1 agonists, such as RO5256390 and ulotaront, could have net effects on DA levels in the synapse which are difficult to predict because they would be expected to decrease DA uptake while simultaneously decreasing DA release by reducing VTA neuron firing.

To add further complication, 2 of the 3 TAAR1 agonists tested, RO5166017 and RO5256390, have direct effects on DAT in that they bind to the RTI-55-/cocaine-DAT binding site and inhibit DA uptake, albeit with low affinity. These data are consistent with previous screens of these TAAR1 agonists at DAT at a single high concentration of drug.^18^^,^^19^^,^^34^ Their direct effects appear more cocaine-like in that they inhibit DAT uptake by binding to the RTI-55/cocaine binding site on DAT. This contrasts with AMPH, which binds to the DAT substrate site and inhibits uptake and promotes efflux of DA. RO5166017, RO5256390, and ulotaront reduce maximum uptake velocity, *V*_max_ (pmol DA/min/mg), which is a function of transport capacity independent of catalytic efficiency/substrate affinity (*K*_m_). The finding that RO5166017 alters *V*_max_ but not *K*_m_ supports the notion that changes in uptake are caused by effects on transporter trafficking rather than posttranslational modifications that alter transporter catalytic efficiency. While we provide support for the hypothesis that TAAR1 agonists affect uptake by modulating surface expression of DAT for RO5166017, there was no statistically significant change in surface expression following RO5256390 and ulotaront in our surface biotinylation experiments.

Notably, while the effects of all 3 TAAR1 agonists on DAT uptake are similarly TAAR1-dependent, they are dissimilar in that RO5166017 increases DA uptake by increasing DAT surface expression, while RO5256390 and ulotaront (and AMPH) decrease uptake.^8^ Although RO5256390 and ulotaront do not appear to decrease DAT surface expression in our biotinylation experiment, it remains possible that they cause a small decrease in surface DAT that we were unable to detect using a biotinylation approach. These results suggest that RO5166017 could be functionally selective in its efficacy at promoting internalization. Furthermore, the fact that RO5166017 increases DAT surface expression, as opposed to having no effect, suggests the possibility that, with respect to the second messengers that drive DAT internalization, RO5166017 could be a weak partial agonist that reduces constitutive activity of TAAR1, which would otherwise act to decrease DAT surface expression in the absence of agonist.^13^^,^^14^^,^^39^ A similar pattern of opposing effects of partial versus full agonists has been previously reported in that TAAR1 partial agonists and full agonists decrease and increase VTA neuron firing, respectively.^21^ Alternatively, RO5166017 may be an inverse agonist that reduces constitutive activity through a key pathway necessary for DAT internalization through which ulotaront and RO5256390 are agonists. Notably, a recent article reported multiple TAAR1 agonists with varying patterns of downstream coupling that were generally broader than that of ulotaront, which was an agonist via G*α*s and an antagonist/inverse agonist via G*α*q and G*α*i.^40^ Also of note is the fact that AMPH promotes DAT internalization by stimulating RhoA via TAAR1–G*α*13 coupling, suggesting the possibility that the 3 TAAR1 drugs tested differ in their ability to promote coupling of TAAR1 to G*α*13-mediated pathways.^8^ Further investigations of the mechanisms by which different TAAR1 agonists influence DAT function will provide important foundational information to guide therapeutic development.

Overall, comparison of the effects of 3 investigational TAAR1 agonists and the TAAR1 agonist AMPH illustrated the unique pharmacology of each at DAT, in contrast to their comparable effect on decreasing VTA DA neuron firing.^21^^,^^22^^,^^41^ AMPH, despite decreasing surface expression of DAT and DA uptake, induced efflux of DA. Notably, all of these effects were TAAR1-dependent, consistent with prior reports. Of the 3 TAAR1 agonists tested, RO5256390 was the most consistent in its activity at DAT in that it directly and indirectly (ie, after pretreatment) reduced DA uptake, an effect that would be expected to counteract the decrease in DA release that results from decreased VTA neuron firing. Interestingly, RO5256390 does not induce motor rigidity, enhances wakefulness, and is procognitive—effects that are suggestive of relatively higher as opposed to lower synaptic DA.^42^ However, pretreatment with ulotaront decreased DAT uptake of DA, without direct binding of ulotaront to DAT. RO5166017 exhibited a complex pattern in that pretreatment increased DA uptake and AMPH-stimulated DA efflux via DAT, while its direct effects were in the opposite direction of the pretreatment effect on uptake (ie, decreased DA uptake).

Together, these findings reinforce the notion that TAAR1 agonists can play a role in DA homeostasis via multiple mechanisms. This includes a less heavily studied role in regulating uptake of DA by regulating DAT, likely by affecting internalization. The most surprising finding is that each of the TAAR1 agonists examined exhibits a unique pattern with respect to direct and indirect effects on DAT, all of which affect the ability of DA to move through the transporter. Given the recent failure of ulotaront in phase III trials despite the promise of TAAR1 agonists in earlier preclinical research, continued research to differentiate the pharmacologic basis for the differences between TAAR1 agonists on DAT and other signaling relevant to DA signaling is warranted.

## Conflict of interest

The authors declare no conflicts of interest.
